# Relationships between Diabetes, Vitamin D Status, Neuropsychological Functioning, and Hispanic Ethnicity in Older Rural West Texans: A Project FRONTIER Study

**DOI:** 10.1002/alz.091599

**Published:** 2025-01-09

**Authors:** Riley McCready, Claudia Morris, Philip Antwi‐Adjei, Mohammad Pourghaed, Felipe Ramirez‐Velandia, Jonathan Kopel, John Culberson, Gabriela Ashworth, Jonathan Singer, Veronica Molinar‐Lopez, Marwan N. Sabbagh, Volker Neugebauer, Boris Decourt, J. Josh Lawrence

**Affiliations:** ^1^ Texas Tech University Health Sciences Center, Lubbock, TX USA; ^2^ Texas Tech University, Lubbock, TX USA; ^3^ Barrow Neurological Institute, Phoenix, AZ USA

## Abstract

**Background:**

Diabetes, a chronic metabolic disease leading to damage to multiple organs and the nervous system, is associated with cognitive decline. Moreover, Vitamin D (VD) insufficiency/deficiency is implicated as a risk factor for diabetes. However, the specifics of the relationships between these variables remain unclear. Previous studies also have suggested that Hispanic individuals have a higher risk of VD deficiency, diabetes, and cognitive decline. We aimed to determine associations between VD level, diabetes, cognitive functioning, and Hispanic ethnicity (HE) amongst a sample of aging, rural West Texans from Project FRONTIER (PF; Facing Rural Obstacles to Health Care Now Through Intervention, Education, and Research).

**Method:**

Data were obtained from a cohort of 292 PF participants (mean age 62.6±11.8, 70.5% female, 40.1% HE). We examined relationships between consensus diabetes diagnosis, blood‐based diabetes‐related biomarkers, VD level, Repeatable Assessment for Neuropsychological Status (RBANS) total score, and HE status. Logistic and linear regression analyses were performed on binary and continuous variables, respectively. We utilized Spearman correlation for bivariate comparisons and Mann‐Whitney U tests for between‐group comparisons.

**Result:**

Regression analyses indicated significant negative associations between VD and HbA1c (p=0.0004), fasting blood glucose (p=0.0003), and consensus diabetes diagnosis (p=0.0060). Regression analyses also indicated significant negative associations between overall cognitive functioning and HbA1c (p=0.0282) and consensus diabetes diagnosis (p<0.0001). A significant positive correlation emerged between VD levels and overall cognitive functioning score (p=0.0439). HE was associated with significantly lower VD levels (p<0.0001), higher HbA1c (p=0.0003), and higher fasting blood glucose levels (p=0.0011) compared to non‐HE participants. HE was associated with lower overall cognitive functioning scores (p<0.0001), indicating greater cognitive impairment.

**Conclusion:**

Our results suggest that, in a rural West Texas cohort, HE is associated with lower VD levels, higher levels of diabetic indicators, and greater levels of cognitive impairment. These disparities are important to consider when investigating areas to improve healthcare in West Texas. Further investigation is necessary to elucidate the connection between VD, diabetes, and cognitive decline in minority, rural populations.

